# Supplementary material for: Cross-Compatibility in Interspecific Hybridization of Different Curcuma Accessions
Source: Plants (Basel). 2023 May 11;12(10):1961. doi: 10.3390/plants12101961 (PMC10220942; doi:10.3390/plants12101961)
Supplement: Supplementary file 1 [file plants-12-01961-s001.zip › Figure S1.pdf]

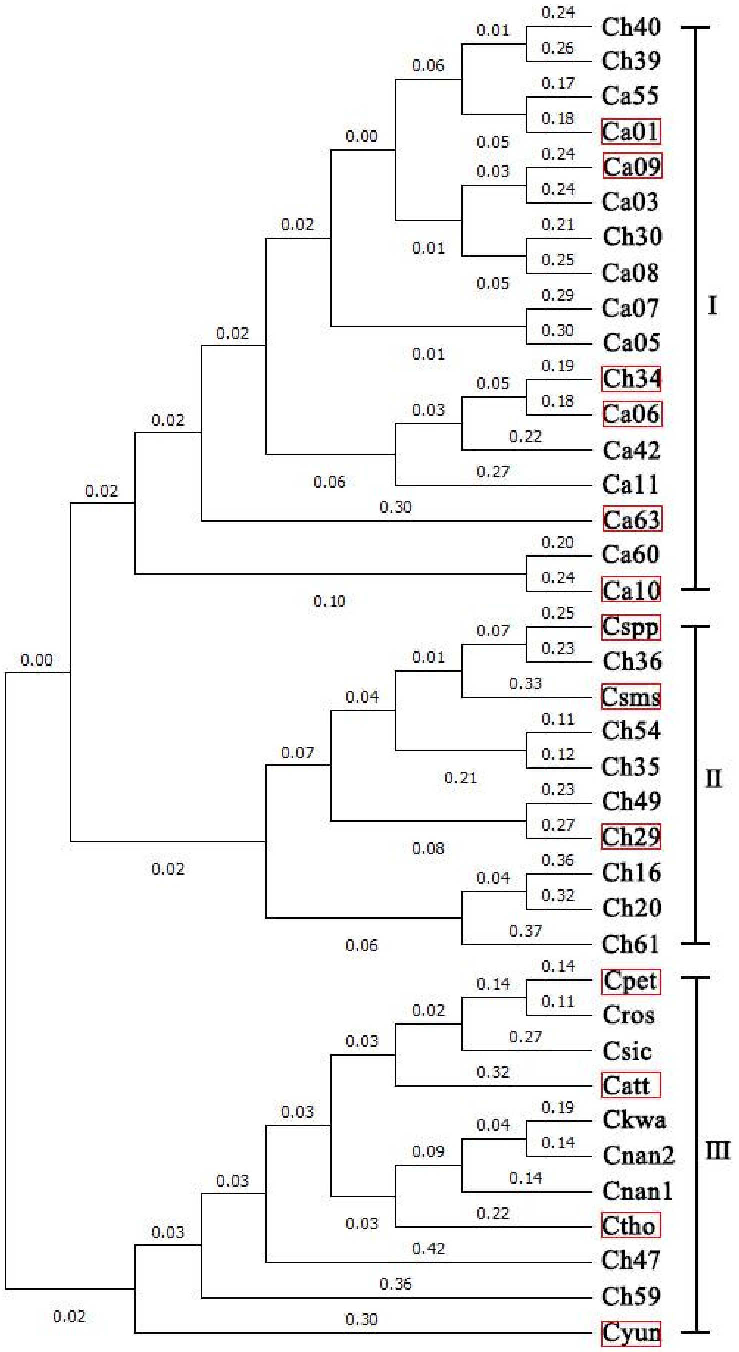

Figure S1. The cluster analysis of 38 *Curcuma* accessions based on 18 EST-SSRs. The samples in the red rectangles were selected as parents for subsequent interspecific crossbreeding.
