# Supplementary material for: Cross-Compatibility in Interspecific Hybridization of Different Curcuma Accessions
Source: Plants (Basel). 2023 May 11;12(10):1961. doi: 10.3390/plants12101961 (PMC10220942; doi:10.3390/plants12101961)
Supplement: Supplementary file 1 [file plants-12-01961-s001.zip › Figure S2.pdf]

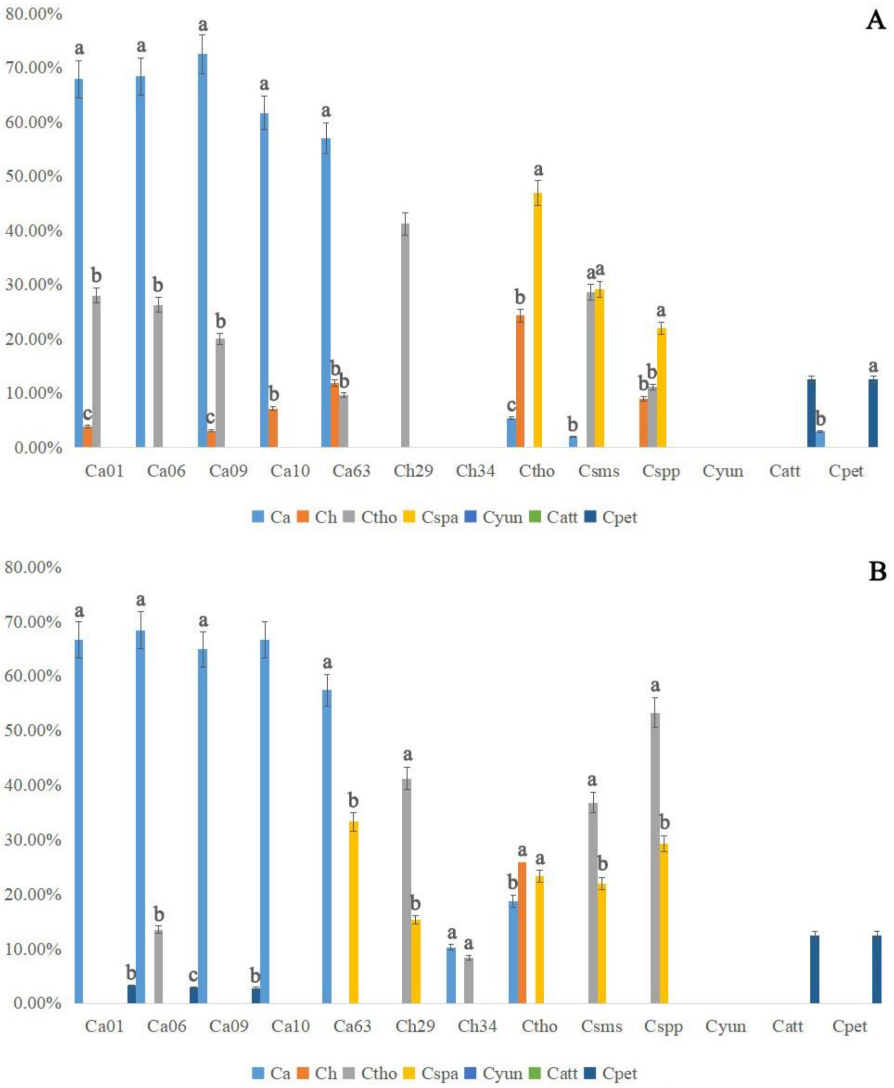

Figure S2. The fruit-setting rates in each hybrid groups of *Curcuma*. (A) 13 accessions in the x-axis as female parent; (B) 13 accessions in the x-axis as male parent. Different lowercase letters represent significant differences ($P < 0.5$).
