# Supplementary material for: Cross-Compatibility in Interspecific Hybridization of Different Curcuma Accessions
Source: Plants (Basel). 2023 May 11;12(10):1961. doi: 10.3390/plants12101961 (PMC10220942; doi:10.3390/plants12101961)
Supplement: Supplementary file 1 [file plants-12-01961-s001.zip › Figure S3.pdf]

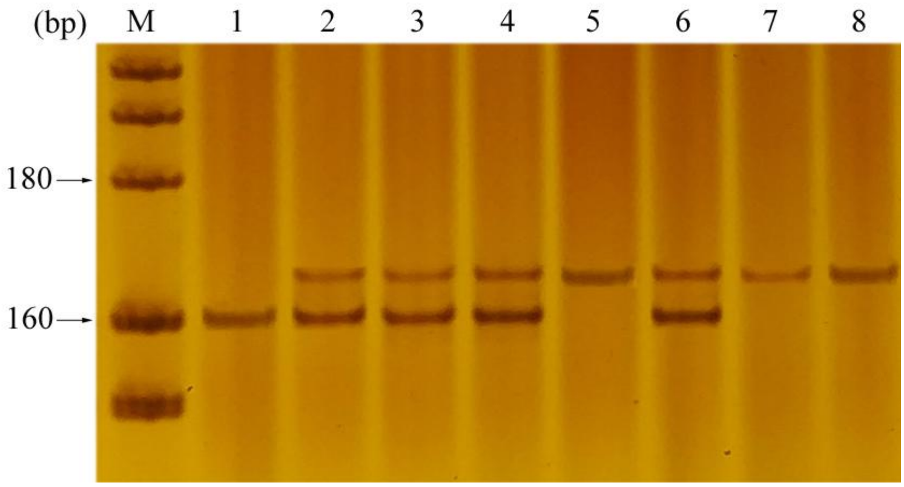

Figure S3. The polyacrylamide gel electrophoresis of JHH10 among 8 samples in Ca01 × Ca10. M represents pBR322 DNA marker; lane 1-2 represent the female and male parent; lane 3-8 represent 6 randomly selected individuals.
