# Supplementary material for: Cross-Compatibility in Interspecific Hybridization of Different Curcuma Accessions
Source: Plants (Basel). 2023 May 11;12(10):1961. doi: 10.3390/plants12101961 (PMC10220942; doi:10.3390/plants12101961)
Supplement: Supplementary file 1 [file plants-12-01961-s001.zip › Table S2.pdf]

Table S2. Polymorphic information of 18 EST-SSRs in 38 *Curcuma* accessions.

Marker name	Sequence (5'-3')	Na	Ne	I	Ho	He	PIC	F
JHH2	F:GATTGGAGGCGGAGAGGAAG<br>R:TTCAGCAGCTCGTCCATGTT	10	3.059	1.542	0.343	0.673	0.683	0.491
JHH7	F:CTTAGCCGCTTCGTGATGGA<br>R:TTGGGCCTCGAAGTGATCAC	9	4.404	1.714	0.375	0.773	0.785	0.515
JHH10	F:CGCGTCAGTGTCTTTACCCT<br>R:CCCTCAGCCGTTCTCTCATC	11	4.685	1.831	0.345	0.787	0.800	0.562
JHH12	F:CGGAGAAGAGAGAGATGGCG<br>R:AACTCCAGCAACGATCCAGG	8	2.695	1.333	0.429	0.629	0.638	0.319
JHH15	F:CGTACCTTGTGGAAGCTGGT<br>R:CTTCTGCTGCTCTGACTGCT	6	4.472	1.605	0.531	0.776	0.789	0.316
JHH21	F:GACTCCAACTCAAGCACTCCA<br>R:TTCCGGGGTCACAATGATGG	9	4.250	1.661	0.971	0.765	0.776	-0.269
JHH34	F:CCCAGTATTCTTTGGGGCGT<br>R:GTCCATTCCCGACCGTCTC	11	4.163	1.814	0.531	0.760	0.772	0.301
JHH37	F:CTCTCGGTGTCGTCACTCC<br>R:CTCTCCGTCCAGTTCGATCG	11	7.847	2.180	0.469	0.873	0.886	0.463
JHH42	F:CCAGAGCGGGGAAGCATATT<br>R:CTGCCACTCTCCACCAAGAA	6	3.696	1.484	0.645	0.729	0.741	0.116
JHH45	F:CTCCGGATCCTTGGAAGACG<br>R:GCGTTCGATTTCTGTGAGCG	6	5.134	1.711	0.889	0.805	0.820	-0.104
JHH48	F:CTCGTCTCGCCTTCTTCCTC<br>R:CTCCTAGTCGCATGCGTCAG	6	2.861	1.265	0.143	0.651	0.662	0.780
JHH53	F:CGTCCGCTTCTCTCTTCCTC<br>R:CAACCTTGAGCGCATACACG	12	3.917	1.757	0.485	0.745	0.756	0.349
JHH54	F:CAGGGGCTCTCAATGTCCTG<br>R:ATCGCAATCCAAGGTGAGCA	15	8.028	2.368	0.706	0.875	0.888	0.194
JHH67	F:TTCCATGGCAGTGGTTGGTT<br>R:ATGAGCTTGGGCAGAGTTGG	12	7.787	2.218	0.690	0.872	0.887	0.209
JHH73	F:GGAAGGGGAAGACTGTGGTG<br>R:CGGTCCATCTTCCAGATCCG	8	3.926	1.684	0.600	0.745	0.756	0.195
JHH84	F:TCTGTCTTCCGTTAGCACTTT<br>R:AAACACCAGTGACCCGTCTC	14	4.330	1.991	0.824	0.769	0.781	-0.071
JHH97	F:TGACCAACGAACGGACTGTT<br>R:CTCGGTTCCACCTCTGGTTC	8	2.593	1.370	0.478	0.614	0.628	0.222
JHH110	F:TTCAAGCTCCATGGCGGAAT<br>R:CTGCGTCCTTGTCAGTGTCT	11	4.148	1.754	0.286	0.759	0.773	0.624
Mean		9.611	4.555	1.738	0.541	0.756	0.768	0.290
